# Thermoanalytical Investigations on the Influence of Storage Time in Water of Resin-Based CAD/CAM Materials

**DOI:** 10.3390/biomedicines9121779

**Published:** 2021-11-26

**Authors:** Martin Rosentritt, Sibylle Schneider-Feyrer, Thomas Strasser, Andreas Koenig, Leonie Schmohl, Alois Schmid

**Affiliations:** 1 Department of Prosthetic Dentistry, UKR University Hospital Regensburg, 93042 Regensburg, Germany; martin.rosentritt@klinik.uni-regensburg.de (M.R.); Sibylle.Schneider-Feyrer@klinik.uni-regensburg.de (S.S.-F.); thomas.strasser@ukr.de (T.S.); alois.schmid@ukr.de (A.S.); 2 Department of Dental Prosthetics and Materials Science, Leipzig University, 04103 Leipzig, Germany; Leonie.Schmohl@medizin.uni-leipzig.de

**Keywords:** Thermogravimetric Analysis, differential scanning calorimetry, dynamic mechanical analysis, resin-based composites, water storage, aging

## Abstract

New resin-based composites and resin-infiltrated ceramics are used to fabricate computer-aided design (CAD) and computer-aided manufacturing (CAM)-based restorations, although little information is available on the long-term performance of these materials. The aim of this investigation was to determine the effects of storage time (24 h, 90 days, 180 days) on the thermophysical properties of resin-based CAD/CAM materials. Thermogravimetric Analysis (TGA), differential scanning calorimetry (DSC) and dynamic mechanical analysis (DMA) were used in the study. TGA provided insight into the composition of the resin-based materials and the influence of internal plasticization and water sorption. Resin-based composites showed different decomposition, heat energy and mechanical behavior, which was influenced by storage time in water. Individual materials such as Grandio bloc showed lower influence of water storage while maintaining good mechanical properties.

## 1. Introduction

Resin-based composites are methacrylate systems, which are highly filled (>75 wt. %) with inorganic substances. With intra-oral digitalization combined with computer aided design (CAD) and computer aided manufacturing (CAM), not only industrially prefabricated ceramics but also polymer-bonded blanks such as resin-based composites and resin-infiltrated ceramics are used today in subtractive process for various indications. In contrast to ceramics, resin-based systems can be applied without additional treatment (e.g., sintering, crystallization, glazing) and therefore have promising high potential in cost- and time-effective applications. One different commercial resin-based material (Vita Enamic) is based on a ceramic network, which is infiltrated with resin. This resin-infiltrated ceramic network provides a higher modulus of elasticity than standard resin-based systems. In comparison to restorative composites, CAD/CAM composites are polymerized in vitro under industrial conditions with high-pressure and high-temperature, improving their mechanical properties [1]. This results in excellent mechanical properties (e.g., E-modulus 10–15 GPa, flexural strength 150–250 MPa); therefore, these materials can be recommended especially for single crowns, veneers, onlays, or smaller anterior fixed partial dentures.

Manufacturers promote resin-based materials for their enhanced edge stability and machinability, especially in thin marginal areas [2,3,4]. Particularly for high occlusal loadings, composites may be preferred in order to preserve antagonistic teeth. Materials with a lower modulus of elasticity are supposed to cause less roughening and less wear of the enamel compared to ceramics [5,6,7,8]. The commercially available materials show strongly varying material composition and resulting mechanical properties [9,10,11,12,13], in vitro behavior [14,15], performance on implants [16,17], fracture toughness [18] and machinability [2,18]. However, the development of materials is progressing very quickly, so that earlier statements are no longer true in some cases for newer materials. All material properties may be influenced by the storage conditions, which may induce water uptake and conversion or relaxing effects [19,20,21,22,23]. Water uptake, swelling, plasticization effects and decomposition are thought to reduce the properties and performance of resin-based materials [24,25,26,27]. Therefore, the comparison after different storage conditions (initial or long-term storage) may help with estimation of these materials’ performance.

Besides standard mechanical tests, thermal analytical methods may help in the temperature- or atmosphere-dependent characterization of resin-based materials. With Thermogravimetric Analysis (TGA), the sample mass is recorded continuously as the material sample heats up. TGA allows, for example, the composition- and conversion-dependent determination of decomposition temperatures, the water and organic as well as inorganic filler content [28,29,30,31]. Differential scanning calorimetry (DSC) may be utilized to determine the endo-/exothermic heat performance of the materials, and may allow for comparison of the composition, reactivity (conversion), glass transition temperature (TG), phase transformation and influence of storage (e.g., water uptake) on the materials [28,29,30,32,33,34]. The areas under the exothermic peak (e.g., crystallization) or endothermic peak (e.g., melting) are proportional to the heat of crystallization or melting. During decomposition, the curve decreases in the exothermic direction. An advantage is that TGA and DSC can be applied on small crushed specimens. Dynamic mechanical analysis (DMA) measures the stiffness and damping of a specimen, and can therefore be used to characterize the material’s elastic or brittle properties as a function of temperature, time, frequency, stress or atmosphere [35,36,37,38,39,40,41,42,43,44]. DMA results are expressed as an in-phase component (storage modulus E’) and an out- of-phase component (loss modulus E’’). The storage modulus (E’) characterizes a sample’s elastic behavior, and the ratio of loss to storage modulus (tan δ = E’’/E’) allows interpretation of the energy dissipation of a material (damping). Long-term elastic and plastic stability are important for dissipating energy and preserving material integrity. Most dental resin-based materials behave neither as perfectly elastic, nor as completely viscous, and therefore are described as visco-elastic materials [45]. All thermal analysis tests share a high reproducibility, which allows interpretation of the results with 2–3 measurements. It is advantageous that precise measurements can be carried out even on small quantities of material.

The aim of this study was to determine the effects of storage time (24 h, 90 days, 180 days) on the thermo-physical properties of resin-based CAD/CAM materials. The hypothesis of this investigation was that the different resin-based composites show different decomposition, heat energy and mechanical behavior, which may be influenced by their storage time in water.

## 2. Materials and Methods

Eleven different resin-based materials were investigated (Table 1) with three different thermal analysis systems (Thermogravimetric Analysis (TGA), differential scanning calorimetry (DSC) and dynamic mechanical analysis (DMA)) (Supplementary Materials). The manufacturer (Netzsch, Selb, Germany) of the thermal analysis equipment provides the temperature accuracy, precision and repeatability of TGA, DSC and DMA in the range of <±0.1–0.3 K, that is, around 0.5%. The resolution was 0.1 µg (TGA), 0.25 µW (DTA) and 0.0005 N and 0.0005 µm (DMA). In addition to the low equipment error, the very high homogeneity of the industrially-produced material samples (no restorations) and the standardized procedure, as well as the low number of influencing factors in contrast to clinical studies, resulted in very high reproducibility of the results. For example, our own TGA tests [31] showed a repeatability of 0.8% for the determination of the filler content of dental materials; these findings can also be transferred to DSC and DMA. The accuracy for peak temperature in ASTM D 3418-99 was given as 1.5 °C and the reproducibility as 2.0 °C. The enthalpy determination had an accuracy of ±5%, according to ASTM E 793-01. Therefore, the accuracy and reproducibility of the instruments, in combination with the test setup (homogeneous sample composition, limited numbers of influence), enabled meaningful measurements even with small sample numbers.

**Figure 1:**
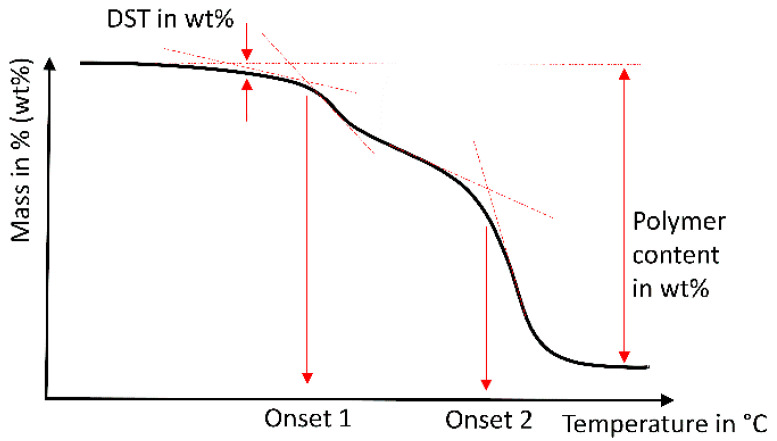
Schematic evaluation of Thermogravimetric Analysis; determination of degradation starting temperature (DST), polymer content (in percentage by mass), and two degradation steps (Onset 1 and 2).

Rectangular specimens (20 mm × 2 mm × 2 mm, *n* = 2 for each experiment) were milled (MCX5, Straumann, Basel, Switzerland) and stored in distilled water (pH 7.4) for 24 h, 90 days or 180 days at 37 °C (mouth temperature, incubator B6, Heraeus instruments, Hanau, Germany) before testing. Both experimental materials were based on UDMA and dimethacrylates. An experimental filler modification was applied to improve the elastic modulus for EXP.1 to a level of 20 GPa (manufacturer’s information), which is clearly higher than that of the other materials. Voco Exp. was based on a slightly-modified monomer formulation of the Grandio bloc, in order to reduce water absorption. This resulted in a clearly reduced water uptake, of only 9 µm/mm^3^. Thermogravimetric Analysis (TGA, TG 209 F3 Tarsus, Netzsch, Germany) was used to weigh specimens (20 ± 5 mg, crushed, *n* = 2 per material) throughout the dynamic heating process (25–600 °C, heating rate 10 K/min, N_2_-atmosphere), which was followed by a static temperature level (600 °C, 25 min). During heating, the materials lost weight in steps; associated temperatures were determined as degradation starting temperature DST (°C; onset temperature, Figure 1). After combustion, the remaining weight in wt. % was determined, indicating the inorganic filler content of the composite.

**Figure 2:**
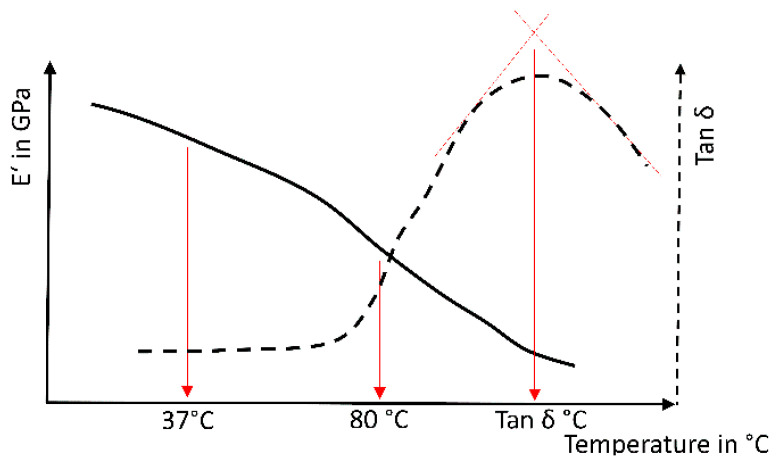
Schematic analysis of a dynamic mechanical analysis; determination of the resulting storage modulus E’ (in GPa) at 37/80 °C and the peak maximum temperature of the tan δ.

Dynamic mechanical analysis (DMA 242 bending mode, Netzsch, Germany) was used to analyze bars (2 × 2 × 10 mm^3^) under controlled mechanical loading (dynamic loading 6 N, static force factor 1.3, amplitude max. 50 µm, multiple frequency 1, 5, 10 and 20 Hz) during a dynamic heating process (20–200 °C, heating rate 5 K/min). The resulting storage modulus E’ (in GPa) (at 37 and 80 °C) and peak maximum temperature (in °C) of maximum tan δ were analyzed at 1 Hz (Figure 2).

**Figure 3:**
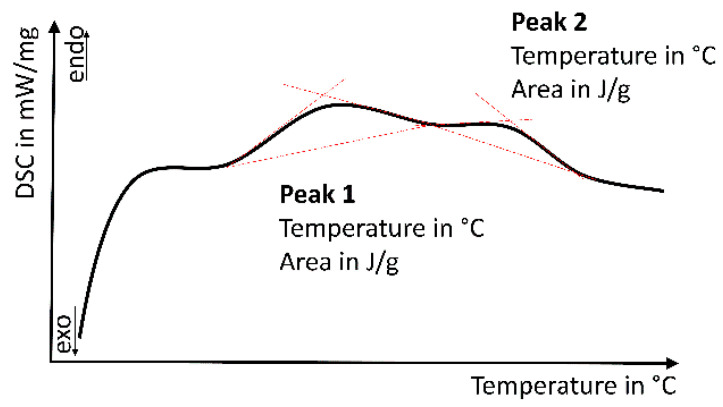
Schematic evaluation of differential scanning calorimetry; determination of two endothermic processes with the endotherm heat flow (area of the peak; in J/g) and corresponding temperatures (in °C).

Differential scanning calorimetry (DSC 204 F1 Phoenix, Netzsch, Germany) was utilized to determine the endo-/exothermic heat (J/g; 40 ± 10 mg, parts of the bars, *n* = 2 per material) during a dynamic heating process (25–300 °C, heating rate 20 K/min, aluminum crucible, N_2_-atmosphere). Data were analyzed as the complex peak analysis of the DSC/temperature curve (peak temperature in °C and peak area in J/mg, temperatures ~80 °C and >150 °C; Figure 3). All thermal analysis evaluation was performed with analysis software Proteus 6.1, Netzsch, Germany.

**Table 1:** Materials, manufacturers, monomers (Bisphenol A diglycidyl ether dimethacrylate = Bis-GMA, Bisphenol A ethoxylate dimethacrylate = Bis-EMA = Bis-MEPP, Diethylene glycol bis(methacrylate) = DEGDMA, Triethylene glycol dimethacrylate = TEGDMA, Urethane dimethacrylate = UDMA) FS: flexural strength in MPa, E modulus of elasticity in GPa; specifications; n.a. = no information available (data according to manufacturer’s specifications or [10,13,27,31,46,47,48,49]).

Product	Manufacturer	Monomers	Mechanics		Filler Content	Water Uptake
			FS	E		
			MPa	GPa	wt. %	µm/mm^3^
Brilliant Crios	COLTENE Holding AG, Altstätten, Switzerland	Bis-GMA, Bis-EMA, TEGDMA [31,49]	198	10.3	70	n.a.
Estelite	Tokuyama Dental, Japan	Methacrylate [48]	225	13.8	70	n.a.
Cerasmart	GC Corporation, Tokyo, Japan	Bis-MEPP, UDMA, Dimethacrylate [31,46,47]	231	12.1 [10]	n.a.	21
Block HC	Shofu Dental GmbH, Ratingen, Germany	UDMA + TEGDMA [27,31,49]	191	9.5 [13]	61	n.a.
Exp1	Experimental	UDMA, dimethacrylate monomer	200	20.0	75	17
Katana Avencia	Kuraray Noritake Dental, Tokyo, Japan	UDMA, dimethacrylate monomer [27,46]	190	12.4	62	n.a.
KZR CAD	Yamakin Co Ltd., Kochi, Japan	UDMA, DEGDMA [27]	235	10.4	65	n.a.
Lava Ultimate	3M Deutschland GmbH, Neuss, Germany	Bis-GMA, UDMA, Bis-EMA, TEGDMA [31,47]	204 [49]	12.7	80	36
Grandio bloc	VOCO GmbH, Cuxhaven, Germany	UDMA + dimethacrylate [31,49]	330	18.0	86	14
Voco Exp	VOCO GmbH, Cuxhaven, Germany	UDMA + di-methacrylate	n.a.	n.a.	n.a.	9
Vita Enamic	VITA Zahnfabrik H. Rauter GmbH & Co. KG, Germany	UDMA + TEGDMA [46,47,49]	150–160	30.0	86	<10

## 3. Results

### 3.1. Thermogravimetric Analysis (TGA)

**Figure 4:**
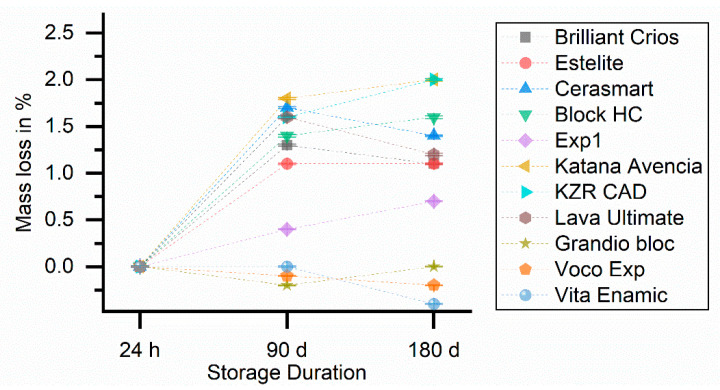
Development of mass loss in wt. % at DST below 280 °C. For better comparison, the values were shifted to originate at 0% at 24 h.

Only Vita Enamic and both Voco materials showed no or only small differences be-tween different storage conditions below 280 °C, indicating limited influence of the storage or saturation already after 24 h. All other materials provided 1−2% differences after 90 or 180 days in comparison to the 24 h values (Figure 4).

Filler content of the investigated systems varied between ~58 wt. % (Katana Avencia) and ~85 wt. % (Enamic, Grandio bloc). The filler content showed no variation due to storage (Table 2). Weight loss at the DST after 24 h was approximately 1 wt. %. Only Vita Enamic, Block HC and Lava Ultimate showed values between 1.7 wt. % and 2.4 wt. %. After 90 days and 180 days at least a doubling of the values took place. Only Vita Enamic and Voco Exp showed no increase due to storage (Table 2).

**Figure 5:**
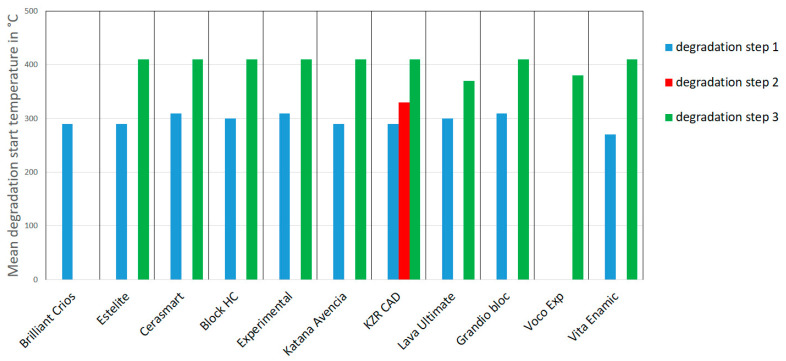
Thermogravimetric analysis (TGA): degradation temperature steps (variance: <2 °C).

The materials provided one (Voco exp, Brilliant Crios), two (other) or three (KZR CAD) onset temperatures. The temperatures were between 280–310 °C and 380–410 °C. Only KZR CAD showed an additional temperature step at ~330 °C (Figure 5).

### 3.2. Dynamic Mechanical Analysis (DMA)

Storage modulus E’ after 24 h varied between 11.7 GPa (Katana Avencia) and 33.4 GPa (Vita Enamic). Storage caused only small changes in E’ for most materials, however, only for Cerasmart, KZR CAD, Block HC and Grandio bloc was a clear decrease (>2.5 GPa) found after 90 or 180 days storage (Table 3). E’ showed a temperature-dependent performance with lower values (ΔE’ ~3 GPa) at 80 °C in comparison to the results at 37 °C (Table 3). Temperature at maximum tan δ varied between 136 °C (Experimental) and 172 °C (Cerasmart). Aging caused a material-dependent shift of the temperatures towards lower values (99.5–154.0 after 90 days, 103.5–165.5 after 180 days) and a broadening of the curve (Table 3). The temperature shift was less pronounced for Cerasmart, Brilliant Crios and Voco Exp. Tan δ for Vita Enamic and Estelite could not be determined because all bars fractured during the testing (Table 3).

### 3.3. Differential Scanning Calorimetry (DSC)

No heat flow could be measured for Vita Enamic. Only Brilliant Crios and the experimental material showed a first endothermic heat flow peak (0.23–0.34 J/g) at around 80 °C after 24 h. After 90 days and 180 days, all materials except Grandio bloc provided an endothermic peak between 0.05 J/g (Estelite) and 0.59 J/g (KZR CAD). After 180 days, values between 0.12 J/g (Voco Exp) and 0.83 J/g (Block HC) were found. Only KZR CAD showed no increase from 90 days and 180 days (Table 4).

Only Brilliant Crios (0.32 J/g), Grandio bloc (0.80 J/g) and clearest Lava Ultimate (3.22 J/g) showed a second endothermic peak (190–264 °C) after 24 h storage. After 90 days only a small second endothermic peak could be found for Brilliant Crios, whereas for Estelite, Cerasmart, Block HC, Katana Avencia Avencia, KZR CAD and Lava Ultimate the second peak was clearly pronounced (>3.5 J/g). After 180 days, only small differences were found for KZR CAD, whereas Brilliant Crios, Cerasmart, Grandio bloc and the experimental material (Exp1) showed formation or increase of a second peak. For Estelite, Block HC and Lava Ultimate the peak after 180 days was less pronounced or disappeared (Katana Avencia). In all others cases, no second endothermic peak could be found (Table 5).

## 4. Discussion

### 4.1. Thermogravimetric Analysis (TGA)

**Figure 6:**
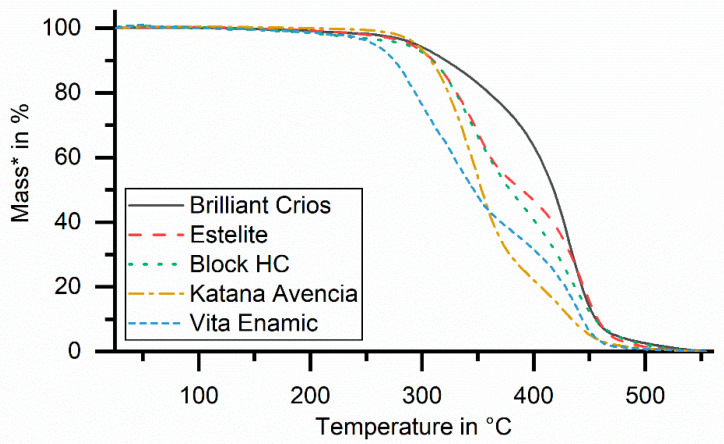
Selected and scaled (* to hypothetical uniform 100% polymer content) TGA curves, based on the measured temperature-dependent mass loss.

The reported water absorption in the polymer networks, which causes plasticizing and softening effects [50] as well as corrosion of fillers [51] or the decay of silanated interfaces between filler and resin, may cause deterioration of the resin-based systems. These small changes might be a sign of molecular degeneration, polymer chain separation, monomer release or water sorption [52]. The constant temperature level of the degradation steps may be a hint towards water uptake; otherwise, with smaller chain length or molecule size, a shift towards lower degeneration temperatures would have taken place. A good relationship between water uptake (Table 1) and TGA results below 280 °C (Table 2) may further support this assumption. Water uptake, as a diffusion-controlled process, is correlated with the type of polymer (hydrophilic parts, e.g., TEGDMA > Bis-GMA > UDMA) and the interface between resin and filler [53]. Similar onset temperatures around 300 and 400 °C might indicate the use of comparable methacrylate systems for the tested materials. There is only limited information about the thermal degradation behavior of the copolymers comprised of different monomers typically used for dental resins in different mixing ratios [54,55]. This makes it difficult to link the TGA results to the exact composition of the CAD/CAM composites. Nevertheless, a pronounced decomposition step at lower temperatures (around 340 °C) can indicate a higher content of defects in the polymer matrix, indicating higher TEGDMA contents [55]. TEGDMA is more likely to produce primary cycle defects because of the less rigid linker between the two polymerized methacrylate groups. Therefore, one would expect a higher TEGDMA content, e.g., for Enamic, Katana, Block HC and Estelite, in contrast to Crios (Figure 6). Furthermore, the existence of two or three steps might be attributed to oligomers, monomer mixtures, or degraded copolymer (methylmethacrylate, phenol). Whether higher temperature levels are an expression of pre-polymerized resin-based filler systems seems unclear, and should be investigated with optical analytics (e.g., scanning electron microscopy). As expected, no influence of storage on the amount of inorganic filler components could be found, indicating no obvious dissolution or leaching of filler components (e.g., glasses). Regardless of the polymer used, the effects of moisture decreased with increasing filler content. In the same way, the modulus of elasticity, the flexural strength, the wear resistance and the hardness increased with the filler content [31].

### 4.2. Dynamic Mechanical Analysis (DMA)

Differences between 10 and 20 GPa were found for E’, which is an indication of different composition, mainly the amount, size, sphericity, and type of filler systems of the materials [23,29,31]. E’-values are in the range of the classical modulus of elasticity (Table 1), standing for the sample’s elastic behavior. Vita Enamic confirmed its special composition polymer-infiltrated ceramic network [56] and manufacturing, with 2–3 times higher E’. Most materials showed a reduction in E’ after 90 days storage, and an even further decrease after 180 days in water. Four materials provided no further decrease or even a small increase, indicating saturation after 90 days storage, or even an already-described increase in brittleness [57]. Vita Enamic again showed some special effects, with decrease after 90 days and increase after 180 days. The performance of the materials was confirmed in most cases for the E’ evaluation at 80 °C as well, however, these values were of a 20–30% lower level, indicating a clear temperature-dependent performance of E’, and therefore of the mechanical properties of the materials in the range of clinical application. These effects should be kept in mind when testing materials under constant laboratory conditions, e.g., at room temperature or body temperature. Comparable storage effects were found for tan δ, with changes of ~1/3 after 90 days storage. The changes were not further developed (or even smaller) with longer storage. Embrittlement may have caused the inability to determine the values for Vita Enamic and Estelite, as these specimens fractured. A broad tan δ peak is typical for highly cross-linked polymers, as their glass-transition is represented not by a point but by a wide range of temperatures. After polymerization, multifunctional monomers contain a heterogeneous network of highly or less densely cross-linked regions. Conditions during polymerization (temperature, pressure) are expected to affect the tan δ temperature location. A broadening of the curve could be interpreted as a sign of post-polymerization and embrittlement of the resin. The shift of maximum tan δ to lower temperatures may be attributed to softening and plasticization (partly expressed in a reduced E’), and in increased damping behavior due to water uptake [58,59]. Again, saturation effects after 90 days were found. DMA results may confirm a reduction in the material’s capacity to elastically and plastically dissipate energy [23], both of which reduce long-term stability, for example under dynamic aging [48]. During heating, the materials expand, increasing their free volume. Bond and side chain movements cause greater compliance of the molecules, and thus the reduction of the elastic modulus. With higher temperatures, the chains in the amorphous regions begin to coordinate large-scale motions, causing a steep decrease in E’. The high conversion of the composites may limit the reaction of trapped radicals or unreacted double bonds, and thus limit post-curing or additional cross-linking effects. Differences in E’ could be attributed to a high percentage of fillers, the combination of different filler sizes, or a high cross-link density [37]. Unexpected effects might also be attributed, for example, to edge effects of the tested bars.

### 4.3. Differential Scanning Calorimetry (DSC)

The results here show no clear picture. Most materials developed an endothermal peak at around 80–90 °C, which was differently expressed (up to 0.65 J/g). Only two materials (Experimental, Brilliant Crios) had a clear peak in this temperature range, even after 24 h storage, which might be an indication of water, smaller additives or monomers with a smaller chain length. Again, these effects may be attributed to water uptake, provided by all materials but Grandio bloc, because for most materials the peak increased with longer storage time (180 days). Only KZR CAD showed a strong unexpected reduction, perhaps due to measurement artefacts. The expression of the peak is strongly dependent on the material, varying between 0 J/g and 0.8 J/g. A further influence of storage is expressed by the development of an endothermal peak above 150 °C (to 211 °C), which was much more strongly expressed (up to 11.8 J/g) in comparison to peaks at lower temperatures. Only three materials (Brilliant Crios, Lava Ultimate and Grandio bloc) showed this peak after 24 h (clearly expressed only for Lava Ultimate), indicating different composition in comparison to the other systems. Only Voco exp, Brilliant Crios, and the experimental material (Exp1) provided no or small peaks. Four materials had a decrease after 180 days, which was clearly contoured for Lava Ultimate. Most obvious was a strong increase for Block HC. DSC results, with changed peak characteristics for different storage times, confirm plasticizing and softening effects on polymer networks, which are caused by solvent uptake [23]. DSC data can mainly be attributed to the resin component, with strong influence from the share of filler; as expected, a higher filler content may cause a reduced share of monomer components, minimizing measurable effects. DSC data allowed no differentiation between resin components and pre-polymerized filler systems. Further tests should deal with the characterization of composite fillers.

### 4.4. General Aspects of Discussion

A feature of all thermal analysis in common is that the stability of resin-based materials can be lost through thermally induced deterioration. This decay is caused by the breaking of molecular chains, and partly by de-polymerization. At lower temperatures the decomposition may be caused by free alkyl radicals, while at higher temperatures chain breaks are caused by pyrolysis. The decomposition temperature of PMMA, for instance, is above 280 °C. A tendency towards de-polymerization occurs with weak C-C bonds in the main polymer chain. This results in changes in the molecular structure (chain splitting and molecular weight reduction, chain cross-linking or chain branching). Functional groups can be converted, e.g., esters can hydrolyze. In addition, low-molecular groups can be split off. When chemical bonds are broken, polymers usually show a reduction in molar mass and thus the formation of low-molecular degradation products. A lower cross-linking density may facilitate water sorption and plasticizing effects. Thermal degradation processes are accompanied by a change in physical properties (e.g., modulus, bending strength) [24,53]. The effects of moisture seem indifferent; a low moisture content in the polymer network had a stiffening effect on the molecular structures, whereas an increased moisture content (0.69–3.0 wt. %) caused a plasticizing effect on the same polymer network. Hydrogen bonding effects arise between the carbonyl groups of the polymer and absorbed water molecules that cause stiffening or plasticizing; bound water may be responsible for different spacing between polymer chains and their resulting deviant mobility [60]. Some questions arise, such as whether reported aging effects by thermal-cycling [23] are just a sign of water storage, and less distinct only due to a short-term storage. It should be kept in mind that the results and deviation of the different TGA methods are influenced by a material’s homogeneity (mixing material or charge effects; small, but local specimens) or testing conditions (atmosphere, heating). Differences in temperature peak location, for example, in the glass transition between individual measuring methods might be attributed to heating rates. Testing under Nitrogen atmosphere avoided oxidation and influenced the combustion. TGA measurements might provide insight into the chemical composition of the resin-based materials and the influence of internal plasticization, water sorption, and degree of cure.

## 5. Conclusions

The hypothesis of this investigation, that the different resin-based composites would show different decomposition, heat energy and mechanical behavior, can be considered confirmed. The results showed that thermal analysis investigation allows for the differentiation and characterization of resin-based systems. All materials showed a more or less pronounced influence from the storage conditions. Saturation effects were found between 90 and 180 days of storage. All materials showed a temperature dependent mechanical performance (storage modulus E’) in the clinical application temperature range between 37 and 80 °C.

The effect of water storage was distinctly pronounced for different materials. Long-term water storage seems recommended for investigating aging effects on resin-based systems. Individual materials, such as Grandio bloc, showed a lower influence of water storage while maintaining good mechanical properties.

## Figures and Tables

**Table 2:** Thermogravimetric Analysis (TGA): Filler content wt. % after decomposition (variance: 0.1−0.4%) and weight% loss (wt. %) at degradation starting temperature DST below 280 °C (variance: 0.1−0.6%).

Property	Filler Content in wt. %			Mass Loss in wt. % at DST below 280 °C		
Storage Duration	24 h	90 days	180 days	24 h	90 days	180 days
Materials						
Brilliant Crios	72.0 ± 0.3	70.8 ± 0.3	71.6 ± 0.3	0.8 ± 0.00	2.1 ± 0.01	1.9 ± 0.01
Estelite	72.4 ± 0.3	71.8 ± 0.3	71.8 ± 0.3	1.1 ± 0.01	2.2 ± 0.01	2.2 ± 0.01
Cerasmart	66.9 ± 0.3	65.6 ± 0.3	66.3 ± 0.3	0.9 ± 0.01	2.6 ± 0.01	2.3 ± 0.01
Block HC	64.1 ± 0.3	63.1 ± 0.3	63.2 ± 0.3	1.9 ± 0.01	3.3 ± 0.01	3.5 ± 0.02
Exp1	78.3 ± 0.3	77.4 ± 0.3	77.0 ± 0.3	0.8 ± 0.00	1.2 ± 0.01	1.5 ± 0.01
Katana Avencia	58.6 ± 0.3	57.5 ± 0.2	57.5 ± 0.2	0.9 ± 0.01	2.7 ± 0.01	2.9 ± 0.02
KZR CAD	69.0 ± 0.3	68.2 ± 0.3	67.8 ± 0.3	0.8 ± 0.00	2.4 ± 0.02	2.8 ± 0.02
Lava Ultimate	75.4 ± 0.3	74.2 ± 0.3	74.8 ± 0.3	2.4 ± 0.01	4.0 ± 0.02	3.6 ± 0.02
Grandio Bloc	84.5 ± 0.3	84.4 ± 0.3	83.9 ± 0.3	1.1 ± 0.01	0.9 ± 0.01	1.1 ± 0.01
Voco Exp	79.7 ± 0.3	80.0 ± 0.3	79.7 ± 0.3	0.8 ± 0.01	0.7 ± 0.00	0.6 ± 0.01
Vita Enamic	85.1 ± 0.3	84.8 ± 0.3	84.7 ± 0.3	1.7 ± 0.01	1.7 ± 0.01	1.3 ± 0.01

**Table 3:** Dynamic mechanical analysis DMA: Storage modulus in GPa at 37 and 80 °C, temperature at max. tan δ (*: fracture < 150 °C during DMA testing, mean and standard deviation).

Property	Storage Modulus E’						Temperature at Max. Tan δ		
		37 °C			80 °C				
Storage Duration	24 h	90 days	180 days	24 h	90 days	180 days	24 h	90 days	180 days
Materials									
Brilliant Crios	12.5 (0.3)	11.9 (0.4)	11.3 (1.4)	9.6 (0.2)	8.7 (0.4)	8.8 (0.2)	168.5 (5.0)	151.0 (1.4)	154.0 (0.0)
Estelite	14.5 (2.0)	14.2 (1.0)	13.7 (0.4)	10.3 (1.0)	8.7 (0.4)	8.5 (0.2)	*	*	*
Cerasmart	12.2 (0.1)	9.2 (0.3)	9.7 (0.1)	9.3 (0.3)	6.8 (0.2)	7.3 (0.1)	172.3 (0.4)	154.0 (4.2)	165.5 (0.7)
Block HC	12.5 (0.2)	9.9 (0.9)	9.3 (1.4)	9.0 (0.4)	6.3 (0.5)	6.0 (0.7)	139.6 (9.0)	107.0 (8.5)	104.0 (1.4)
Exp1	16.0 (2.5)	19.5 (2.8)	14.9 (0.9)	11.9 (1.6)	12.2 (0.2)	11.1 (0.9)	136.5 (0.7)	110.5 (2.1)	109.0 (1.4)
Katana Avencia	11.7 (0.1)	11.0 (0.6)	10.4 (0.4)	8.6 (0.1)	7.0 (0.3)	6.9 (0.1)	161.5 (7.8)	109.0 (2.8)	112.5 (14.8)
KZR CAD	13.7 (0.7)	10.2 (2.3)	10.3 (2.2)	10.0 (0.5)	7.5 (0.6)	7.8 (0.3)	165.5 (2.1)	113.0 (12.8)	118.5 (3.5)
Lava Ultimate	15.3 (2.5)	15.8 (0.3)	16.5 (2.0)	12.0 (1.7)	10.9 (0.1)	12.9 (0.7)	138.0 (2.8)	99.5 (0.7)	103.5 (4.9)
Grandio Bloc	20.0 (1.3)	16.4 (2.6)	17.2 (0.5)	16.7 (0.5)	13.9 (0.8)	15.3 (1.7)	166.5 (4.9)	132.5 (12.0)	141.0 (1.4)
Voco Exp	17.0 (1.1)	16.9 (0.7)	15.5 (0.5)	14.0 (0.9)	15.5 (0.4)	12.3 (0.1)	160.5 (0.7)	150.0 (4.2)	155.0 (7.1)
Vita Enamic	33.4 (0.2)	30.0 (2.4)	38.5 (5.6)	31.5 (3.1)	25.2 (1.8)	30.9 (4.5)	*	*	*

**Table 4:** Differential scanning calorimetry (DSC): endotherm heat flow (first peak; J/g) and corresponding temperature at ~80 °C after 24 h, 90 days and 180 days; *: peak not to identify, mean and standard deviation.

Storage Duration	Peak Area in J/g			Temp in °C
	24 h	90 days	180 days	
Materials				
Brilliant Crios	0.23660 (0.02121)	0.38260 (0.064347)	0.50215 (0.00092)	85
Estelite	*	0.05265 (0.02213)	0.40640 (0.01386)	80
Cerasmart	*	0.16745 (0.02694)	0.19565 (0.08323)	90
Block HC	*	0.40175 (0.04999)	0.83390 (0.05812)	80
Exp1	0.34270 (0.01994)	0.35290 (0.02800)	0.52955 (0.07743)	83
Katana Avencia	*	0.54060 (0.08683)	0.64890 (0.01994)	82
KZR CAD	*	0.59945 (0.10642)	0.34835 (0.01817)	82
Lava Ultimate	*	0.25045 (0.01464)	0.33255 (0.02807)	80
Grandio bloc	*	*	0.20485 (0.01266)	85
Voco Exp	*	0.07382 (0.00231)	0.12440 (0.07382)	90
Vita Enamic	*	*	*	*

**Table 5:** Differential scanning calorimetry (DSC): heat flow (second peak; in J/g) and corresponding temperature (in °C) after 24 h, 90 days and 180 days (*: peak not to identify, mean and standard deviation).

Storage Duration	24 h	Temp	90 days	Temp	180 days	Temp
Materials		°C		°C		°C
Brilliant Crios	0.3272 (0.0288)	264	0.2292 (0.1303)	217	4.3811 (4.4586)	216
Estelite	*	*	6.5555 (1.4601)	194	1.0066 (0.1250)	186
Cerasmart	*	*	3.5270 (2.5017)	205	6.1885 (1.5846)	196
Block HC	*	*	11.8245 (3.5150)	154	5.2800 (3.8622)	151
Experimental	*	*	*	*	18.6200 (0.9758)	*
Katana Avencia	*	*	7.2140 (4.1379)	178	*	176
KZR CAD	*	*	5.1800 (1.5895)	192	6.8525 (0.3217)	201
Lava Ultimate	3.2225 (0.0233)	212	9.2805 (0.7771)	172	4.3740 (0.5670)	161
Grandio Bloc	0.79775 (0.0777)	190	*	*	5.8925 (1.1801)	*
Exp2	*	*	*	*	*	*
Vita Enamic	*	*	*	*	*	*

## Data Availability

The data presented in this study are available in the article.

## Supplementary Materials

The following are available online at https://www.mdpi.com/article/10.3390/biomedicines9121779/s1, Attached are photos of the technical equipment and of the samples and equipment that were used. In addition, we have added DSC, TGA and DMA pictures of one material as an example.
