# Supplementary figures and images for: Thermoanalytical Investigations on the Influence of Storage Time in Water of Resin-Based CAD/CAM Materials
Source: Biomedicines. 2021 Nov 26;9(12):1779. doi: 10.3390/biomedicines9121779 (PMC8698771; doi:10.3390/biomedicines9121779)

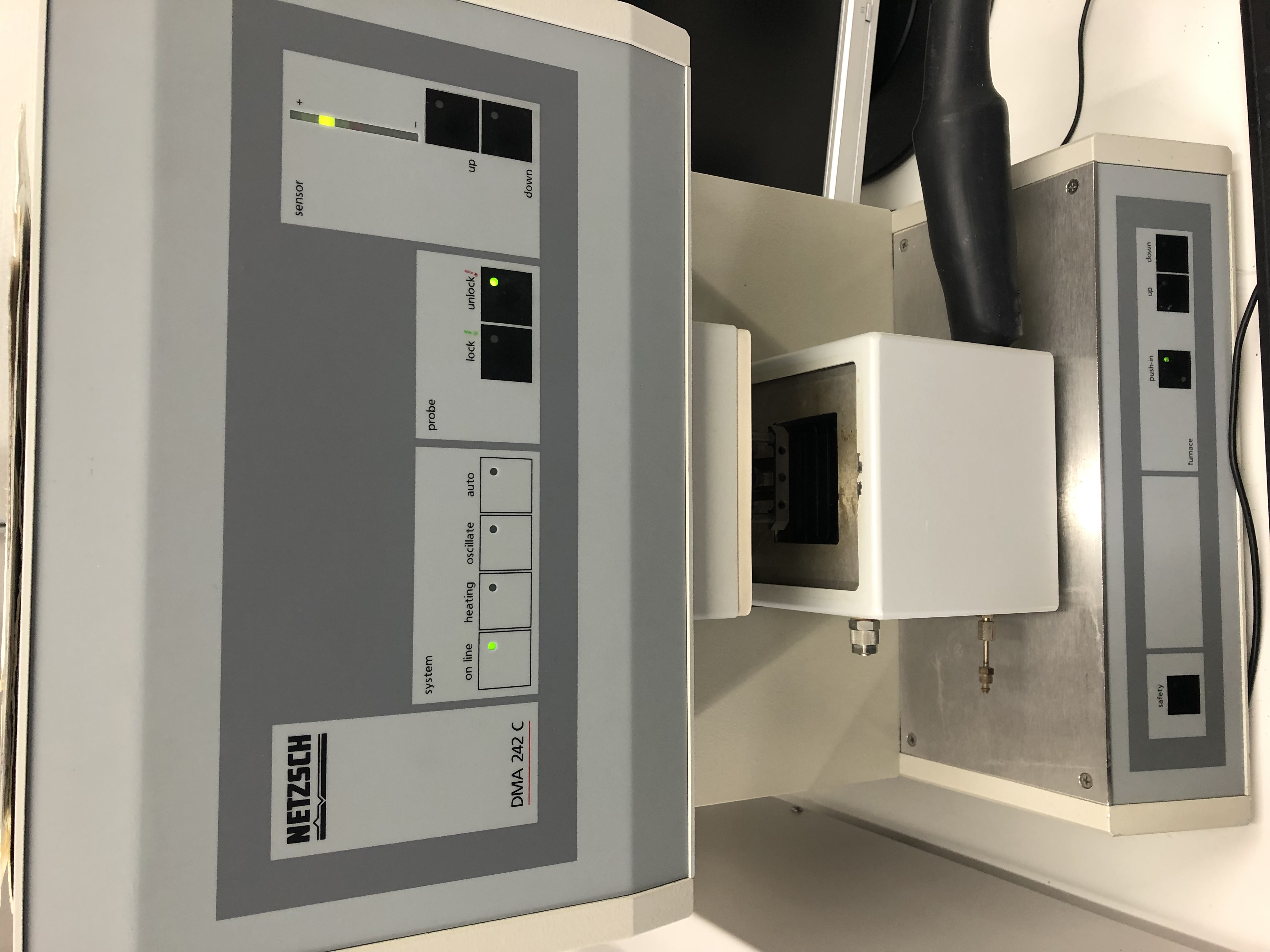

Supplement: Supplementary file 1 [file biomedicines-09-01779-s001.zip › DMA.jpg]

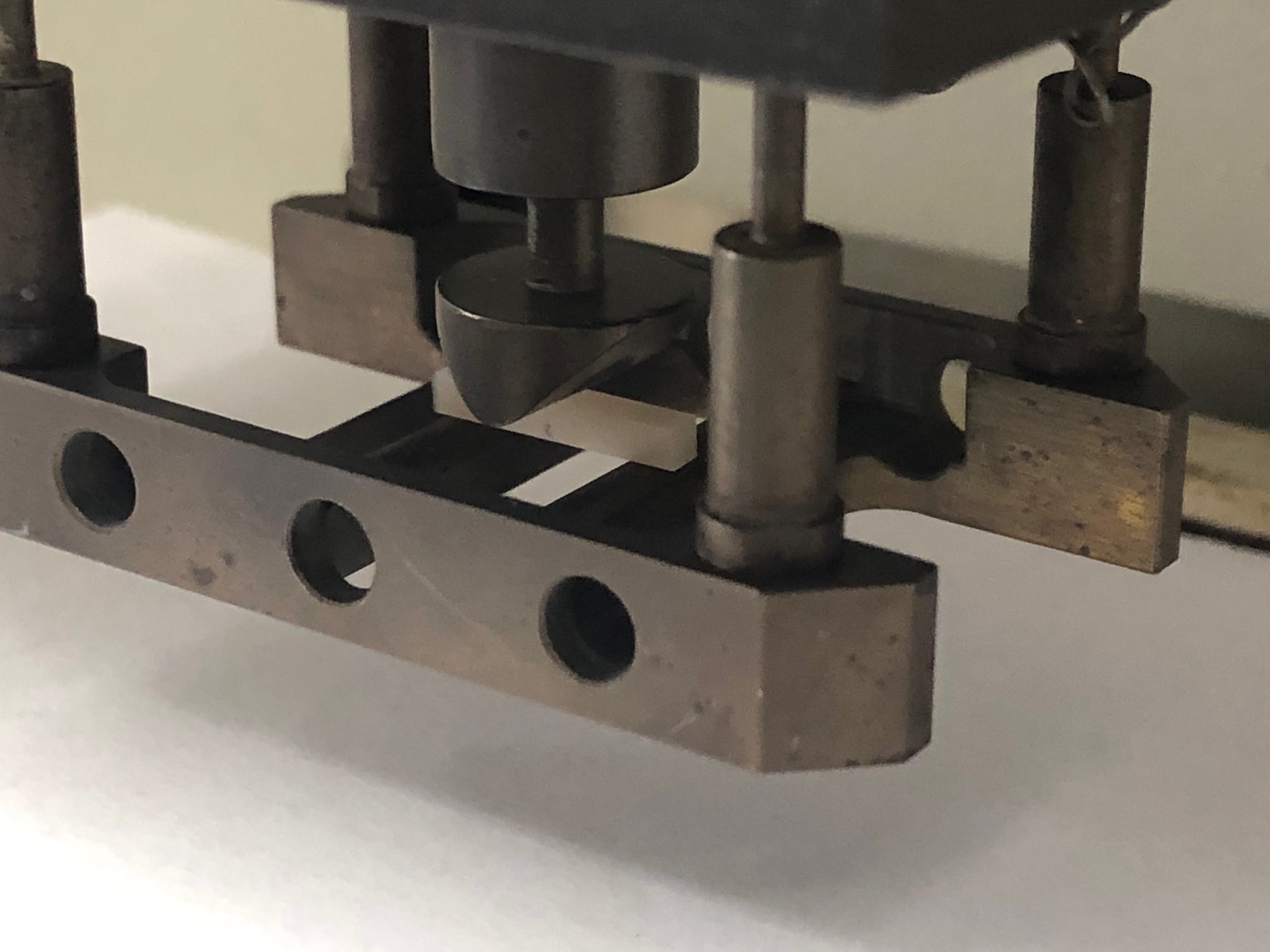

Supplement: Supplementary file 1 [file biomedicines-09-01779-s001.zip › DMA_Sample.jpg]

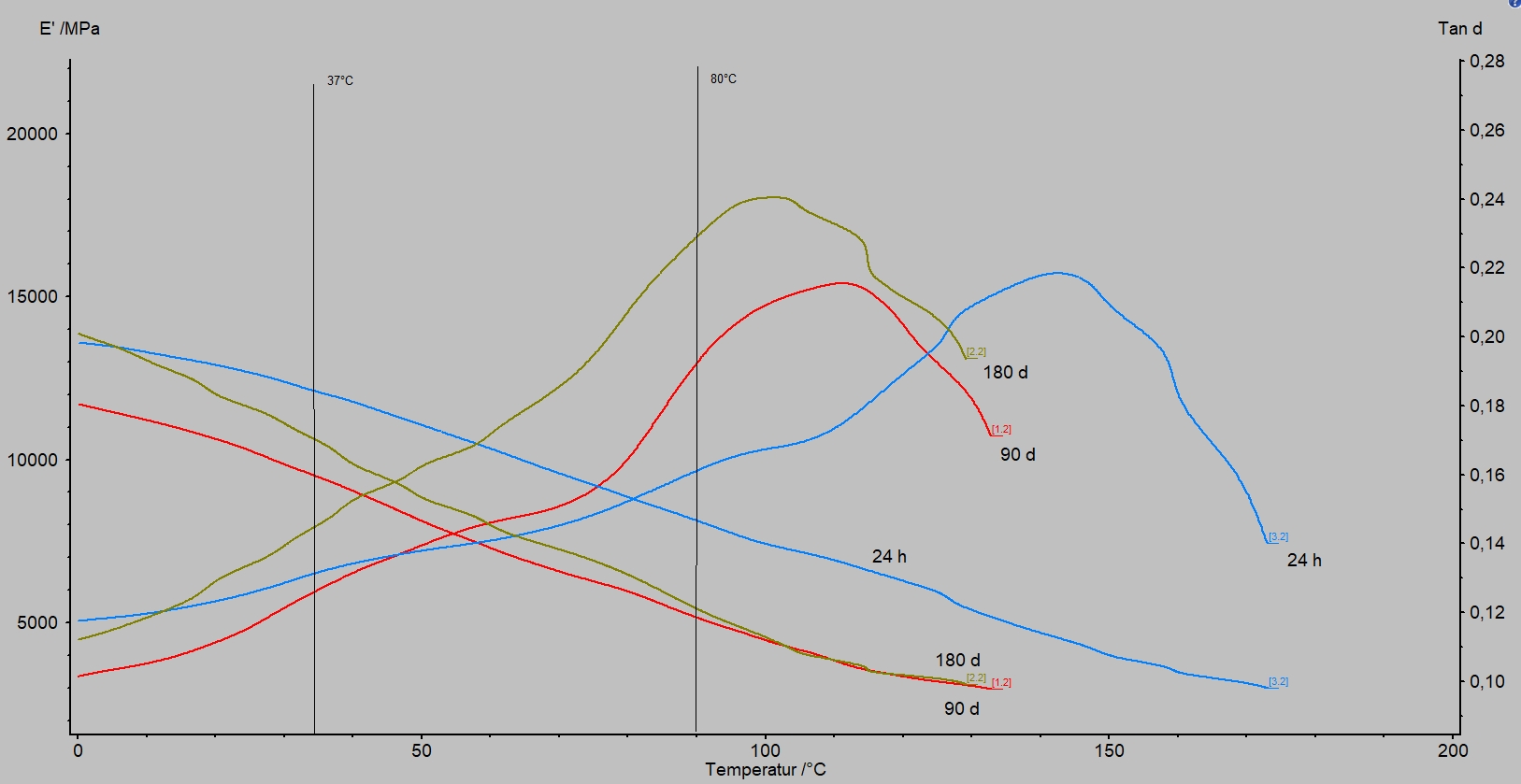

Supplement: Supplementary file 1 [file biomedicines-09-01779-s001.zip › DMA__example.jpg]

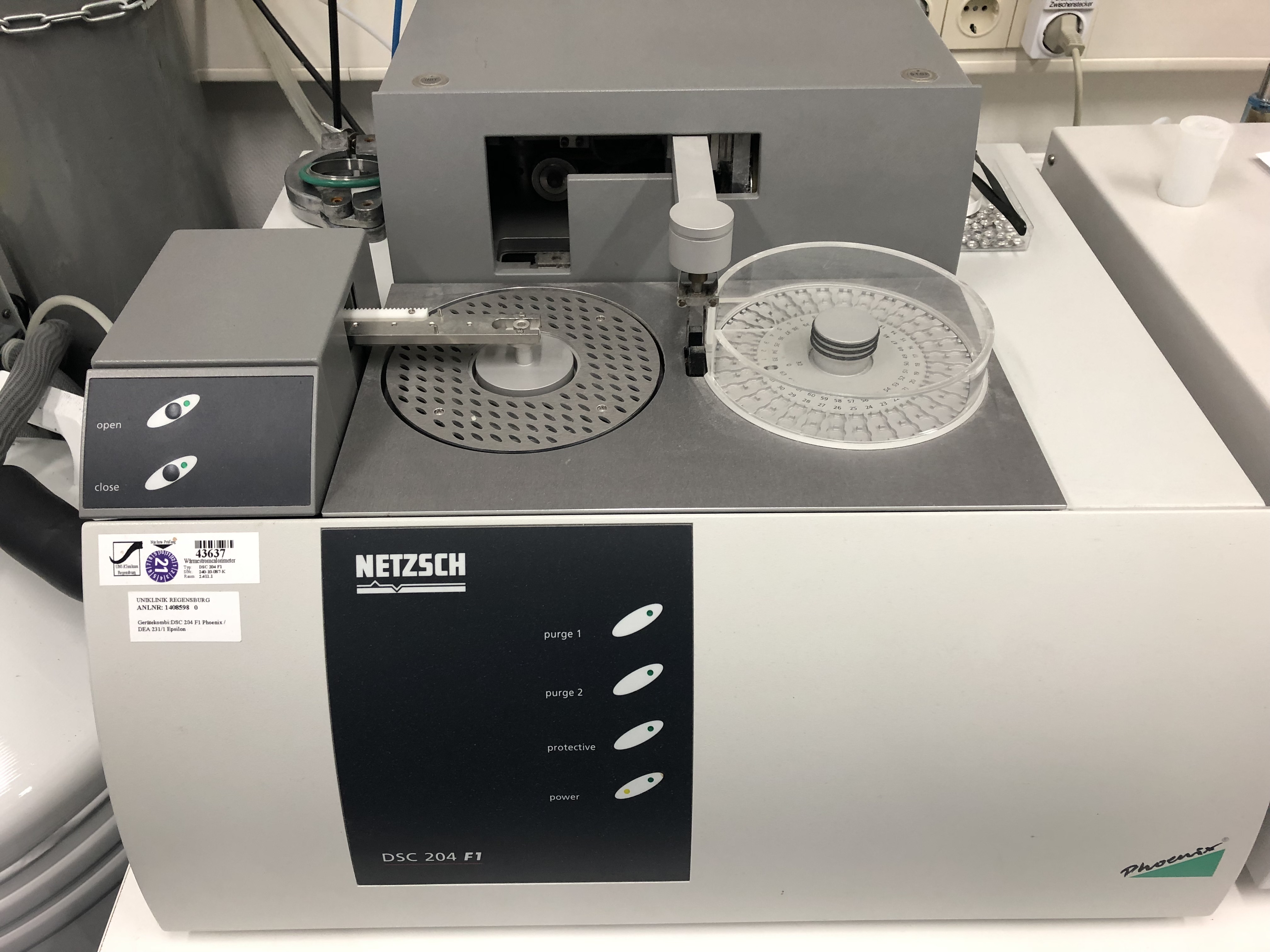

Supplement: Supplementary file 1 [file biomedicines-09-01779-s001.zip › DSC(1).jpg]

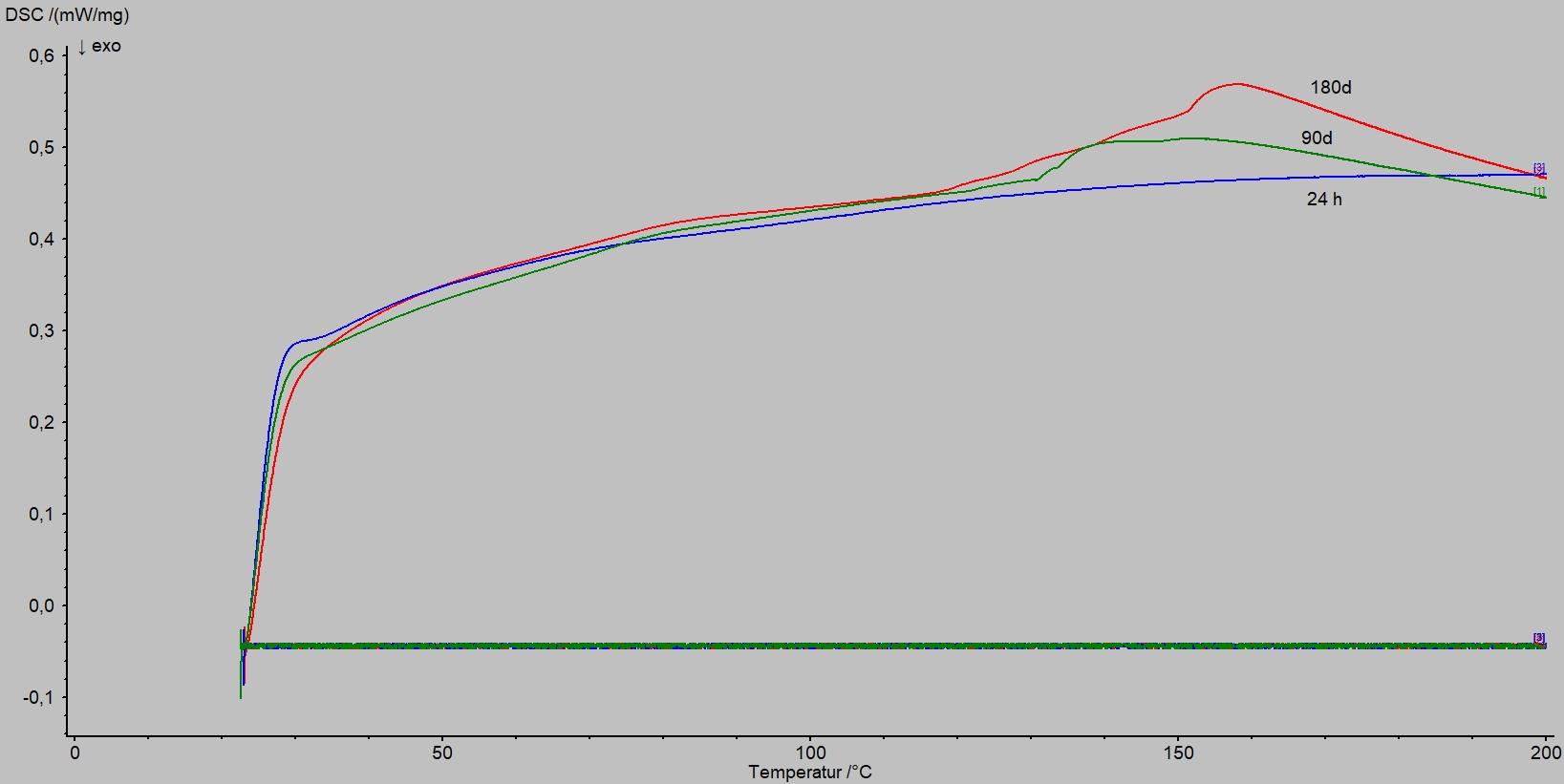

Supplement: Supplementary file 1 [file biomedicines-09-01779-s001.zip › DSC_example.jpg]

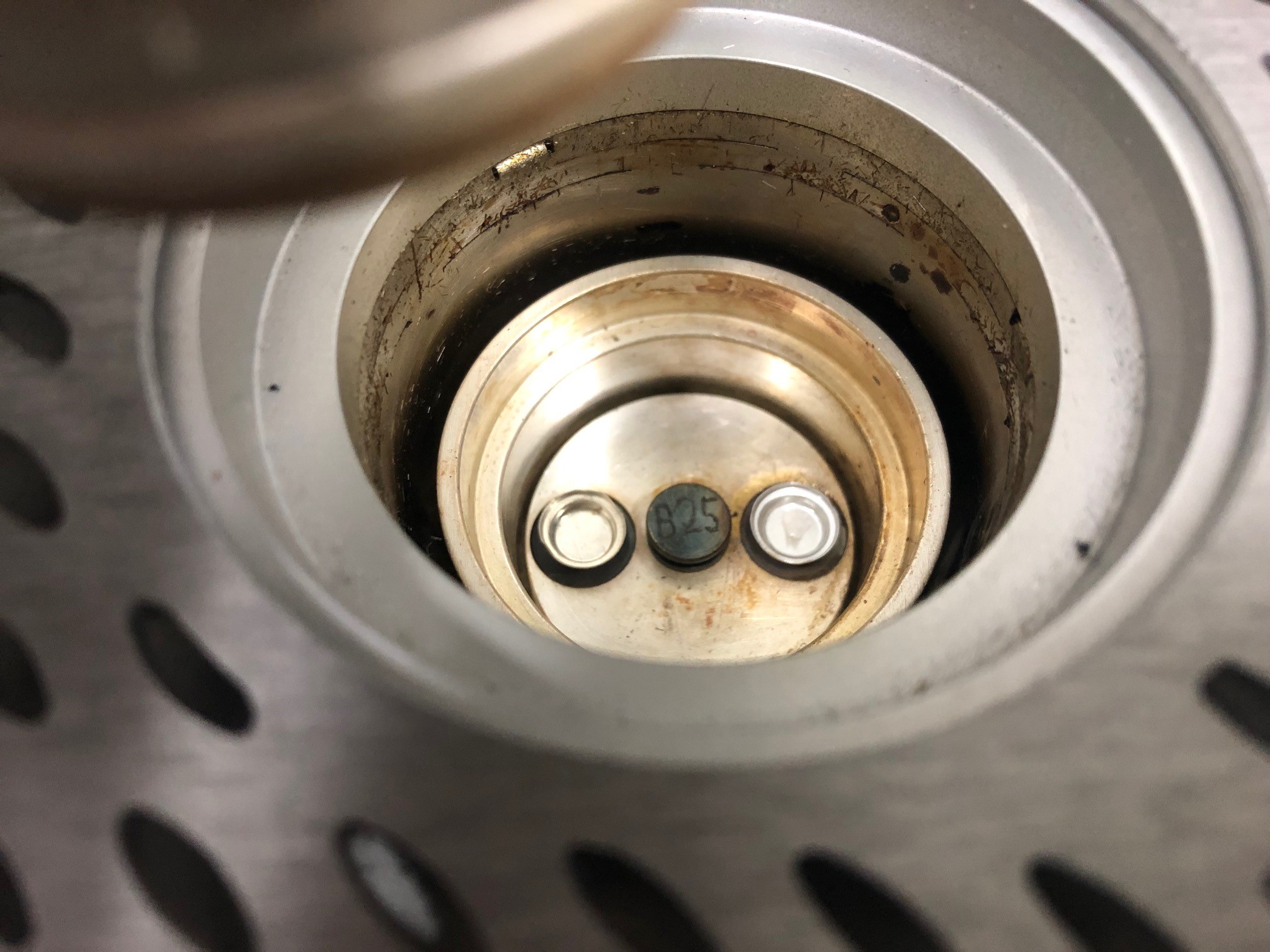

Supplement: Supplementary file 1 [file biomedicines-09-01779-s001.zip › DSC_sample.jpg]

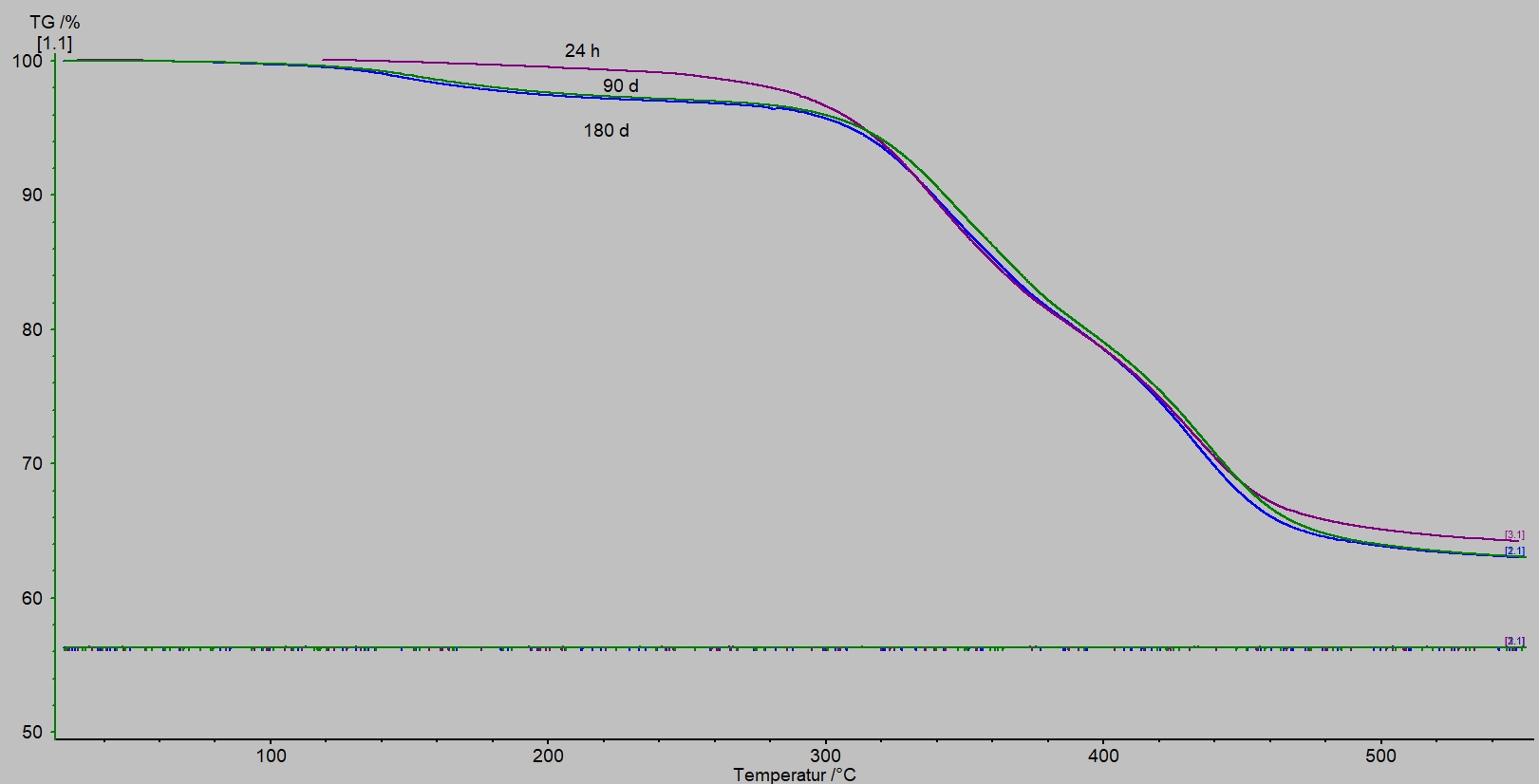

Supplement: Supplementary file 1 [file biomedicines-09-01779-s001.zip › TGA_example.jpg]

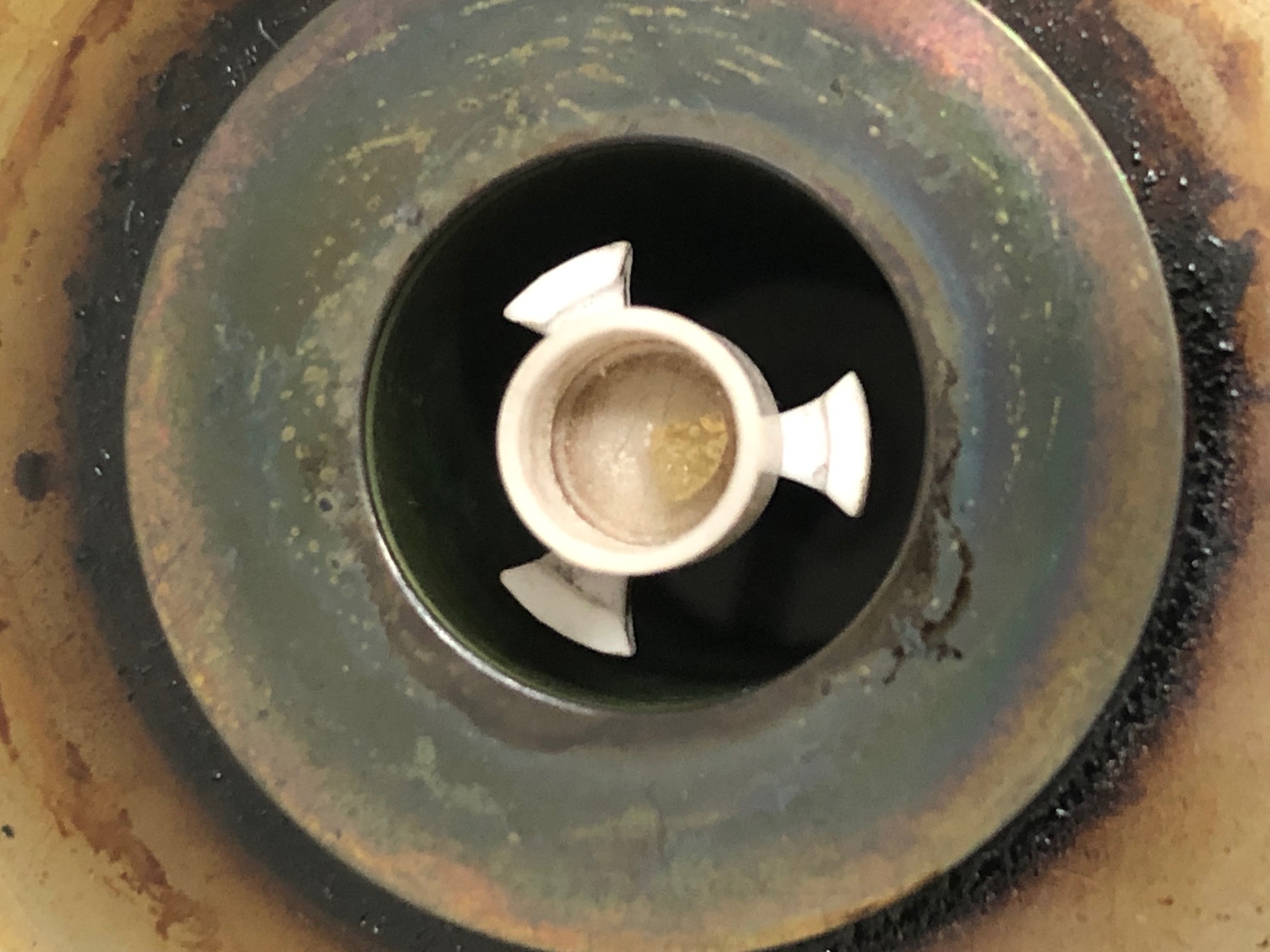

Supplement: Supplementary file 1 [file biomedicines-09-01779-s001.zip › TGA_sample.jpg]
